# From inaction to integration? Media coverage of climate-health policy approaches and solution target in global north-south countries

**DOI:** 10.1016/j.joclim.2025.100619

**Published:** 2026-03-21

**Authors:** Rabia Qusien

**Affiliations:** Rabia Qusien, postdoctoral associate, The Alliance for a Sustainable Future, The George Washington University

**Keywords:** Climate change, Climate and health communication, Media coverage, Government policy response, Inaction, Sustainable response

## Abstract

**Introduction:**

Climate change has become a major health concern worldwide, while the policy response to these challenges exists in silos. In democracies, the media have the power to influence the policy agenda by highlighting certain issues and they act as a key platform for debating the political, social, and health implications of climate change. This research reveals how policy discourse is communicated to the public, directly influencing the political feasibility and social acceptance of climate-health policy actions.

**Method:**

This research presents an analysis of media coverage of government approaches and solutions across the Global North (the United States and the United Kingdom) and the South (Pakistan and India). A quantitative content analysis of two newspapers from each country, from 2015 to 2024, was conducted to analyze the government's approach.

**Result:**

Media coverage of climate and health reveals that inaction prevails across all selected countries, while sustainable policy responses are less emphasized. Denial of the climate-health connection is more prevalent in the Global North, particularly in the United States. After 2019, media coverage showed a reactive response to the issue. Media in all countries focused on government-led initiatives to manage health issues caused by climate change (47.4 %). Even the media in the Global South missed the opportunity to highlight global cooperation for climate-health action.

**Conclusion:**

These findings suggest that a country-specific anticipatory approach for integrating climate and health considerations into policy and communication across regions is needed to mitigate the health impacts of climate change.

## 1 Introduction

The higher frequency and intensity of extreme weather events, such as heatwaves, floods, wildfires, droughts, and storms, have a significant impact on human health and wellbeing. Climate change impacts a wide range of diseases, from non-communicable diseases to vector-borne diseases [1]. Ambitious policy action can improve public health and conserve the environment [2], such as greenhouse gas reduction strategies, which have co-benefits to safeguarding health for the most vulnerable segments of society, and are a key adaptation goal in climate action [3]. However, policy response to climate-health challenges is slow.

Climate change has become increasingly politicized, with denial and delay discourses slowing policy action [4,5]. This politicization is compounded by temporal challenges in climate policy, as politicians operating in short election cycles are reluctant to address long-term climate costs [6]. There is a political commitment to address the health impacts of climate change, yet climate and health policies exist in silos [7]. Complex health issues requiring multisectoral approaches are poorly contextualized in policy [3]. In a polarized environment, an integrated approach for robust policy response is necessary for climate-health action. Despite the growing evidence linking climate change to its health impacts, little is known about the health effects of climate change and associated mitigation actions in various contexts [7].

### 1.1 Climate and health communication

Communication researchers emphasize health reframing of climate change for better public engagement [8,9,3] because health-framed climate information may motivate a broad segment of the public across the political spectrum for collective action [3]. As the intensity and frequency of extreme weather events increase, the government and media are expected to effectively communicate climate-health information to the public [10]. For example, during the heat dome in Canada in 2021, media (especially digital news platforms) extensively covered this extreme weather event with quotes from government sources. Despite more coverage, only 23 % of articles include heat-related health implications, aligning with the government bulletin. Moreover, these messages often included “contradictory content, inconsistent language, or incorrect advice” [6].

Previous research shows a limited focus on public health implications of climate change [11]. Two studies of five US newspapers yielded a corpus of 270 and 354 news stories on climate change and health issues between ^January 1, 2011^ and ^December 31, 2012^, and between January 1, 2007, and December 31, 2008, respectively [12,13]. Similarly, a study from New Zealand confirmed a total of 523 climate-health related articles appeared in newspapers in New Zealand from ^January 1, 2001^, to ^April 14, 2016^ [11]. Although there is an increase in climate-health reporting, it remains insufficient and is experiencing a downward trend. For example, the number of news stories on the health relevance of climate change decreased by 10 % between 2022 and 2023 in English, Chinese, German, Portuguese and Spanish newspapers [14]. Additionally, there is a decline in government mentions of climate and health in their annual UN General Debate statements [14]. Moreover, media framing of climate change often foregrounds the mitigation frame (how to slow down climate change and reduce future impacts). It places less emphasis on preparing for and adapting to new climatic conditions [15]. Despite media coverage trends and its framing strategies, the role of the media is essential in the formulation and implementation of climate change policy, especially when policies aim for a coordinated and multisectoral response [16].

Media framing may influence governance and policy outcomes through the salience of the issue, policy agenda setting, and mobilization of action [17]. Scholars have widely studied the influence of the news media on public opinion. Agenda-setting theory suggests that an issue becomes important to the public when it becomes salient in the media [18,2]. Additionally, media and policy do not operate in isolation from one another — each shapes and is shaped by the other in an ongoing, reciprocal relationship [19]. A higher volume of media coverage of climate change is associated with greater support for climate action [19]. However, despite more support for the climate emergency narrative among scientists, politicians, and activists, media coverage of policy action remains low, and the US government's approach remains reactive [17]. Media coverage may shape public understanding of policy challenges, either reinforcing sectoral silos through fragmented reporting or promoting integration by framing issues as interconnected. However, limited research has examined how news media representations of government responses to climate-health issues are covered across different national contexts.

News media analysis offers a critical lens to examine how governments publicly communicate and legitimize their climate-health policy approaches, revealing patterns of policy integration, responsiveness, and priorities across different national contexts. However, news media coverage of climate-health policy may not reflect the full scope of governmental action, as news media, guided by journalistic norms and values, shape what is newsworthy and how to frame policy actions, often prioritizing novelty, conflict, and crisis. Yet, this research will focus on the mediated representation of climate-health policy, rather than actual government policy.

Previous research in the area of health impacts communication in relation to policy has largely emphasized high-income nations in the Global North [2,20]. However, due to geography, exposure, and sensitivity to health impacts, certain populations are substantially more vulnerable to the health harms of climate change [3]. For example, the public in the Global South is particularly vulnerable to the health impacts of climate change, while the Global North is most responsible for climate change impacts. This study examines the government's approach to climate and health through a comparative analysis of news media coverage in four countries: the USA, the UK, Pakistan, and India. This research will show how communication varies across different media systems and policy contexts, as well asacross Global North vs.South countries.

This study explores the following research questions.

RQ1: To what extent do newspapers portray government approaches to climate-health issues across regions?

RQ2: How do news media representations of governments' approaches to climate-health issues across regions vary over time?

RQ3: Who is held responsible for solutions in climate and health news coverage across different national media contexts?

## 2 Materials and methods

A quantitative content analysis was conducted to analyze media reporting of the government's response to climate-related health harms in the Global South (Pakistan and India -- highly vulnerable to climate change) and North (USA and UK -- vulnerable to climate change, but major contributors to the issue of climate change) [21].

### 2.1 Data collection

Two leading newspapers from each country are selected because they potentially can set the agenda for other media and influence the policy agenda [21]. The selected papers are considered trustworthy sources of information by the respondents. According to a Reuters digital report *The New York Times* and *USA Today* (USA) and *The Guardian* and *The Times* (UK) are considered trustworthy news sources by almost 50 % of the public in their respective countries, and 65 % of Indians trust *Hindustan Times* and *The Times of India* (India) [22] Similarly, *Dawn* and *The News* (Pakistan) are credible news sources in Pakistan [23]. Additionally, the news media are a dominant source of information for the public on climate change [24]. The time frame for this study spans from January 2015 to December 2024, as the Paris Agreement was reached in 2015 and The Lancet Countdown on health and climate was established. Since 2015, the UN has held an annual Conference of the Parties (COP) and The Lancet has published its annual report each year. The LexisNexis Uni was accessed to collect data using the following query.

• ("climate change" OR "global warming" OR "climate crisis")
• W/5
• ("health" OR "public health")

This search resulted in 2500 stories, which were downloaded and saved as Word files. The researcher read the headline and the first paragraph of the collected articles to remove duplicates and articles containing keywords without direct reference to the health implications of climate change.

### 2.2 Coding framework

After data cleaning, a final corpus of 941 stories (which included news stories (640), op-eds (149), editorials (18), and features (134)) was analyzed using the coding framework below. Three dimensions of analysis were conducted in this study (see Table 1).

**Table 1:** Analytical dimensions guiding the study.

Analytical dimension	Description
Coverage level	Frequency of climate health policy representation across newspapers and years
Government approach	Reactive, Delay, Inaction, Denial, Sustainable
Solution target	Government led, integrated, specialist and external, No solution

Quantitative content analysis was conducted to explore the level of media coverage of government policy responses to the health implications of climate change, the types of government approaches discussed in the media discourse over time, and where the media discourse directed solutions.

Government approach: This variable encompasses various government positions on climate-related health harms, ranging from inaction, denial, and delay to reactive and sustainable approaches (see Table 2).

**Table 2:** A list of government approaches in climate-health coverage.

Government approach	Definition	Examples
Inaction	Failure to take adequate climate-health action [4,34]	“Republicans might as well deny climate change if they don't plan to address it; Mere acknowledgement that the environment is in peril without a plan to mitigate it is a huge oversight” [35].
Deny	Rejection or dismissal of the scientific consensus on anthropogenic climate change and its health harms [4].	“One faux executive explains how oil companies ''invested in political campaigns'' as the screen shows President Trump calling human-caused climate change ''a hoax.'' He lauds the president for ''rolling back all those fossil fuel regulations.''[9]”
Delay	Discourse that acknowledge the reality of climate change and its impact on health but justify inaction or insufficient efforts to address it [4].	“Andrea Rodgers, a lawyer for the plaintiffs, noted that the administration regularly points to its progress in promoting fossil fuels. (It recently referred to methane as ''freedom gas.'') Yet, she said, ''as the delays continue, the government-created public health disaster gets worse.''”[36]
Reactive	Action taken after an event or issue occurred [37] to address health implications of climate	“President Donald Trump repeated his claim this week that Democratic leaders in California deserve blame for the fires, having failed to clear leaves and dead trees from forest floors. Wally Covington, professor of forestry at Northern Arizona University, agreed that forests have become overgrown and need to be thinned, but "not with a lawn rake."”[38]
Sustainable	A proactive approach to mitigate and adapt to climate change’s health impacts to build resilience [37]	“Under this programme, an environmental health cell has been set up in the state and a survey is being conducted on heat-related disorders as well as respiratory infections in the most polluted cities in the state. In connection with the Climate Change and Human Health programme, various workshops will be organized to create awareness among the general public and the health department officials and staff [39]”. “Boosting tree cover in urban areas through sustainable and community-driven urban forestry initiatives has been made a central part of the country's largest afforestation program, launched last year as part of Prime Minister Imran Khan's vision for the Clean and Green Pakistan programme, [40]”

To understand policy discourse, it is also important to understand whether solutions or climate action targeted in the media discourse were government-led action (both local and national government action), integrated solutions (solutions in which communities and institutions act together), specialized and external solutions (focused on medical practitioners, science and technology related solutions, and global cooperation), or whether no solutions were mentioned.

### 2.3 Data analysis

A total of 200 news items, representing content from each of the eight newspapers in the sample, were selected to assess intercoder reliability. This sample of news items was coded by an independent research assistant trained in content analysis.^1^ After coding 20 stories, both coders met to review and resolve discrepancies before finalising the codebook. Cohen’s kappa was calculated overall across all categories for each variable, yielding the intercoder reliability *k* = 0.8 for the government approach and *k* = 0.87 for the solution target variable. To analyze the coded data, descriptive statistics, cross-tabulations, chi-square, and Cramér's V tests were conducted.

## 3 Results

RQ1: Analysis of media coverage shows that US media (especially *NYT*) covered government approaches most frequently (76.1 %), followed by the UK (62.2 %, especially *the Guardian*), Pakistan (56.1 %, especially *Dawn*), and India (42.5 %), respectively (see Table 3). There is a statistically significant association between the country and the level of media coverage of government approaches towards climate-health issues, χ²(3, *N* = 941) = 59.44, *p* < .001, Cramér's *V* = 0.25 (small to medium size effect). A statistically significant relationship was also found between the selected newspapers and government approach, χ² (7, *N* = 941) = 106.294, *p* < .001, *V* = 0.34, indicating a medium effect size. While statistically significant differences exist across countries and newspapers, the small-to-medium effect sizes indicate that cross-national patterns share important commonalities.

**Table 3:** Level of government approach towards climate-health covered in the corpus. (Note percentages represent proportion of articles within each newspaper/country that mentioned government approach (Present) vs. did not mention it (Absent)).

Country name	Newspaper	Absent (n,%)	Present (n,%)
USA	*NYT*	46 (18.3 %)	205 (81.7 %)
	*USA Today*	25 (54.3 %)	21 (45.7 %)
	Total	71 (23.9 %)	226 (76.1 %)
UK	*The Guardian*	56 (29.9 %)	131 (70.1 %)
	*The Times*	37 (62.7 %)	22 (37.3 %)
	Total	93 (37.8 %)	153 (62.2 %)
Pakistan	*Dawn*	40 (36.7 %)	69 (63.3 %)
	*The News*	47 (52.8 %)	42 (47.2 %)
	Total	87 (43.9 %)	111 (56.1 %)
India	*Hindustan Times*	55 (57.9 %)	40 (42.1 %)
	*The Times of India*	60 (57.1 %)	45 (42.9 %)
	Total	115 (57.5 %)	85 (42.5 %)
Total		366 (38.9 %)	575 (61.1 %)

**Fig. 1:**
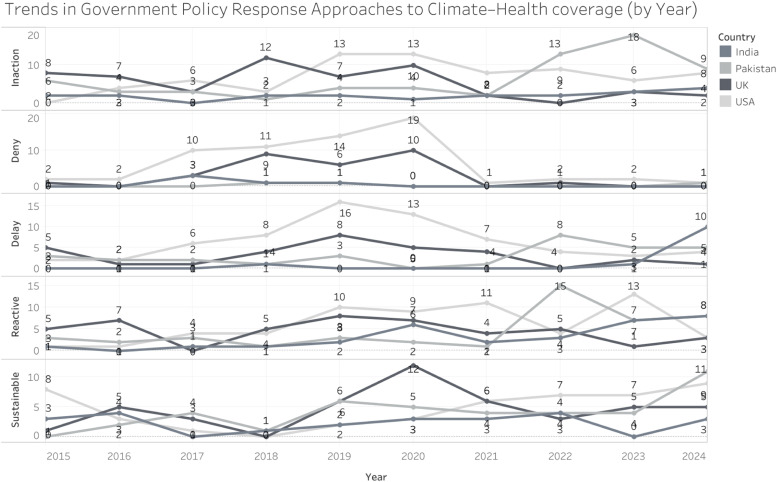
Media coverage of government approach across countries from 2015–2024.

RQ2: Across all four countries, newspaper coverage portrays government responses to climate-health issues as predominantly characterised by inaction, with solution-oriented reporting remaining comparatively limited. The reactive government approach appeared to increase across regions after 2019, which may be associated with a higher number of extreme weather events resulting from climate change. Overall, there was a decline in mentions of government denial and delayed tactics over time. There were specific patterns across countries (see Fig. 1 in Section 3). However, there are important regional patterns of coverage, such as Pakistani news media showing decreasing coverage of government denial and reactive responses over time. There was a spike in inaction and a reactive policy discourse in the media after 2022, which can be attributed to the super flooding in 2022 and the increasing frequency of heatwaves each year. Surprisingly, inaction peaked in 2023, suggesting media criticism intensified after the disaster highlighted preparedness failures. Additionally, a rise in coverage of sustainable policy approaches in 2023–2024 may be due to the UN COP27’s “Loss and Damage” momentum led by Pakistan [25] On the other hand, Indian news media indicate a consistent improvement (with more focus on solutions and sustainable coverage) in the government's response to climate-related health harms, and minimal denial coverage throughout, indicating relatively stable policy commitment across election cycles. While media discourse around inaction and delay aligns with the COVID-19 crisis and heatwave episodes, revealing gaps in preparedness. In the Indian context, PM Modi's re-election campaign in 2019 coincided with a sharp rise in denial and reactive discourse, suggesting that electoral politics may influence the tone of climate-health coverage during this period.

Media coverage of government inaction on climate-health issues in the UK intensified between 2018 and 2020, a pattern that may reflect the displacement of climate policy from the political agenda during the Brexit period, despite growing pressure from advocacy organisations such as Extinction Rebellion [26]. However, after 2020, these patterns experienced a downward trend, which may be linked to the UN COP26 in Glasgow in 2021 (which was scheduled for 2020 but delayed due to COVID-19). The US news media discussed government inaction, denial, and delaying tactics. The most relative to other countries, especially during Trump's first term (2017–2020), reaching its highest point of 19 articles in 2019, before dropping precipitously to near-zero following Biden's inauguration in 2021. This shift demonstrates how media coverage of the government's approach to climate-health policy reflects the administration’s policy position. Overall, there appears to be mixed progress, with improvements in sustainable approaches across regions, but inaction also remains a dominant trend (see Fig. 1 in Section 3).

RQ3: In the analysis of media coverage of solutions, government-led initiatives received the highest coverage across all selected countries (47.4 % of the entire sample), while 17.7 % of the coverage did not discuss any solutions (see Table 4). There is a notable presence of integrated solutions (26.1%), which involve various segments of society collaborating towards climate-health action. On the other hand, very few articles focused on international cooperation, especially in the Global South (12.1 % and 1.5 % in Pakistan and India, respectively). There is a statistically significant relation between the type of solutions reported and the countries in which the media reporting occurred, χ²(9, *N* = 941) = 30.962, *p* < .001, *V* = 0.10 (small effect). This small effect size indicates that solution targeting patterns are relatively consistent across countries, with government-led solutions dominating universally (47.4 %), despite some variation, such as the notably low coverage of external/specialist solutions coverage in India (1.5 %).

**Table 4:** Solutions coverage across selection countries. (Note: Percentages represent proportion of articles within each country discussing each solution type).

Solution target	USA (n,%)	UK(n,%)	Pakistan(n,%)	India (n,%)	Total(n,%)
No solution	42 (14.1 %)	36 (14.6 %)	41 (20.7 %)	48 (24 %)	167 (17.7 %)
Government led	140 (47.1 %)	130 (52.8 %)	78 (39.4 %)	98 (49 %)	446 (47.4 %)
Integrated solutions	84 (34.1 %)	56 (22.8 %)	55 (27.8 %)	51 (25.5 %)	246 (26.1 %)
Specialized and external	31 (10.4 %)	24 (9.8 %)	24 (12.1 %)	3 (1.5 %)	82 (8.7 %)

## 4 Discussion

This study presents an analysis of media coverage of government approaches in four countries (India, Pakistan, the USA, and the UK) regarding the health implications of climate change and solution targets. The study's findings reveal temporal patterns in the coverage of climate-health policy responses. Governments are portrayed as inactive in all selected countries (see Fig. 1 in Section 3), suggesting that climate-health issues are consistently framed through a lens of policy inadequacy and fragmentation, despite the climate vulnerability of all countries [27]. While statistically significant news coverage differences exist across countries and newspapers (*V* = 0.25–0.34), the prevalence of media coverage of inaction across all regions (see Fig. 1 in Section 3) and the consistent dominance of coverage of government-led solutions (*V* = 0.10) suggest that climate-health policy fragmentation is a systemic challenge transcending national contexts.

Media coverage explicitly reported on coordination challenges underlying these patterns. For example, large metropolises (i.e., Delhi and Mumbai) lack a clear climate-health resilience plan due to the multiplicity of problems and the multiple authorities responsible for managing these challenges. Several departments, such as public health, water and environment, energy, and social justice, need close coordination, but they work in silos. Notably, the factors driving this discourse also largely fall outside the remit of health ministries alone, underscoring the need for a more coordinated action across government sectors [28]. Beyond inaction, the coverage also points to the absence of a coordinated, multisectoral approach to climate-health challenges, with sources emphasising that short-term, uniform government responses are insufficient and that effective public health and emergency measures require sustained cross-sectoral engagement [29]. Collectively, these examples highlight institutional fragmentation and present climate-health challenges primarily as governance failures rather than offering integrated framings that transcend administrative boundaries. Additionally, the consistent coverage of inaction may itself become a barrier to policy integration by normalising stagnation.

News analysis shows that media portrays governments in all selected countries, especially those in the Global South, as adopting a reactive, event-driven approach and crisis-dependent policy responses (e.g., reactive to the 2022 floods in Pakistan). These findings reflect journalistic norms that skew reporting toward conflict and disasters or crisis rather than providing a comprehensive account of actual government actions. Disasters or crisis moments can create a policy window with temporarily weakened institutional barriers, in which media attention can highlight more sustainable policy options. Yet, media coverage in such events prioritizes emergency response rather than systemic integration, potentially wasting opportunities for discourse on lasting policy change. Additionally, the presence of a reactive approach and delay tactics in news media suggests that policymakers operating in short election cycles are reluctant to address long-term climate-health issues [21]. Therefore, when media discourse emphasized climate-health issues, it focused on government reaction instead of proactively reporting risks, preparedness, and the need for a coordinated policy response and systemic change essential for climate-health action [22]. Hence, there is the challenge of long-term integration thinking for a sustainable response to addressing climate-health challenges. The prevalence of media coverage of inaction and reactive government approaches across countries validates the policy integration literature, which suggests that institutional silos create systematic barriers to cross-sectoral coordination.

Beside patterns of inaction and reactive responses, regional differences in media framing also emerged. The prevalence of denial of climate-health issues in the USA may reflect climate skepticism in the Global North (where fossil fuel industry and their front groups create barriers to meaningful climate action) [8,30] compared to the Global South (where the public and the media believe in anthropogenic climate change and understand that there is minimal scientific uncertainty) [31,32]. Additionally, the spikes in coverage are consistent with each government's approach to the issue, such as higher level of denial from 2017 to 2020 in the USA. However, the media coverage of climate-health policy reveals a decrease in denial over time, which supports emerging literature [33]. Taken together, these patterns suggest that government communication strategies remain inadequate for promoting sustained climate-health integration, as media coverage across all four countries continues to be dominated by inaction and denial rather than solution-oriented policy discourse.

Analysis of RQ3 shows additional communication gaps. For example, the literature suggests harnessing community engagement for climate-health action [28]. The findings of our solution type analysis (Section 3.3) provide empirical evidence of fragmentation in climate-health discourse: the dominance of government-led solutions (47.4 %) and limited coverage of integrated, multi-stakeholder approaches (26.1 %) demonstrate how media coverage reinforces sectoral rather than integrated thinking about climate-health responses. This shows a gap in how the health implications of climate are communicated to audiences. However, there is a need to co-create feasible climate-health solutions involving local communities [28]. Additionally, the media discourse in the Global South provided less space for specialized responses and global cooperation, despite the country-specific needs for international collaboration and institutional capacity building. This may limit public understanding of the need for global cooperation frameworks to address the health harms of climate change. To better adapt to these challenges, experts, especially health professionals, need to be involved in climate-health policymaking to ensure health equity [28]. Overall, an ambitious policy response is necessary to mitigate the health impacts of climate change globally, particularly in protecting vulnerable populations in the Global South.

There are study limitations, such as the use of news media coverage as a proxy for policy discourse; future research can conduct policy discourse analysis. The sample of this study was limited to English-language newspapers. It included only two newspapers from each country, which may not represent the full breadth of media sources in all selected countries. Future research can analyze different media, particularly those operating in multiple languages. Keyword choices in the data collection may have excluded some relevant articles. Finally, the corpus for this study comprises a mix of news articles, op-eds, editorials, and features, which may influence the framing patterns.

This research provides the following practical implications for journalists, policymakers, and scholars.

• **Journalists**

*Journalists should integrate the health implications of climate change policies into their reporting, foregrounding preventive measures and an equity narrative, especially during extreme weather events, rather than focusing solely on damages or statistics (i.e., a disaster frame). For example, when covering a heatwave, journalists should not report only the death toll but also preventive measures, identify the vulnerable population, and highlight the availability of cooling centres.*

• **Policymakers**

*The government should integrate health considerations into their climate policy and communication, emphasising the health co-benefits of climate action. For example, the transition to low-carbon infrastructure should be framed not as an emission reduction measure, but as a means to reduce respiratory illnesses by improving air quality.*

• **Scholars**

*Researchers should further investigate how regional and non-English media frame the linkage between climate and health policies, as well as their influence on policy legitimacy and public understanding.*

## 5 Conclusion

News media coverage of policy responses does not adequately support the level of action needed to protect against the projected climate-health risks. This study identified specific institutional and communication barriers for climate-health policy, such as the media's emphasis on reactive response and government-led action for addressing the challenges. An increased emphasis on media coverage of climate change impacts on health can engage the public and foster policy actions to mitigate the health consequences of climate change. These findings suggest a more integrated approach to policymaking that ensures an equitable response and prioritizes institutional capacity building, particularly in the Global South, given the region's high climate vulnerability. The news media need to reframe the issue in their coverage to support anticipatory policy action, multi-level solutions, and global cooperation narratives. To achieve this, there is a need to create climate change and health literacy among policymakers and communicators.

Codebook

Name of newspaper

1. Dawn
2. The News
3. Hindustan Times
4. The Times of India
5. The Guardian
6. The Times
7. New York Times
8. USA Today

Name of country

1. Pakistan
2. India
3. UK
4. USA

Year

1. 2015
2. 2016
3. 2017
4. 2018
5. 2019
6. 2020
7. 2021
8. 2022
9. 2023
10. 2024

1. Government approach

**Table:** 

Government approach	Definition	Example
Inaction	Failure to take adequate climate action [4,34]	“THE Obama administration's whiplash decision last week to allow oil and gas companies to drill along a wide area of the Atlantic Coast is a big mistake. The facts support a ban on offshore drilling not only in the wilds of Alaska – as the administration has announced – but also along our densely populated, economically vibrant and environmentally diverse Eastern Seaboard. The BP Deepwater Horizon disaster should remind us that the benefits of drilling do not outweigh the threat to local economies, public health and the environment when an inevitable spill occurs [41]”.
Deny	Rejection or dismissal of the scientific consensus on anthropogenic climate change [4].	“WASHINGTON — The Environmental Protection Agency plans to change the way it calculates the health risks of air pollution, a shift that would make it easier to roll back a key climate change rule because it would result in far fewer predicted deaths from pollution, according to five people with knowledge of the agency’s plans.” [30]
Delay	Discourse that acknowledge the reality of climate change but justify inaction or insufficient efforts to address it [4].	“A panel of lawmakers also warned this week that “killer heat waves” would become more common in Britain because of climate change. The Environmental Audit Committee warned that heat-related deaths would triple to 7000 every year by 2050 if the government does not take action.”
Reactive	Action taken after an event or issue occurred [37]	“According to a press release issued by CISS, the HEC chairman regretted that the current national policy on climate change and the strategy for its implementation were weak, inconsistent, incoherent and reactive. The policy, he observed, looks to be a 'long list of unprioritized measures and agencies' and without any 'rationale and targets.' He said it was not surprising because of these policy weaknesses that targets were not met” [38]. “The heat has also exacerbated London’s toxic air-pollution levels, prompting the mayor, Sadiq Khan, to issue a warning on Thursday. “The heat, combined with London’s toxic air, a lack of cloud cover and emissions traveling from the continent means I am triggering a ‘high’ air pollution alert today, for tomorrow, under our comprehensive alert system,” Mr. Khan said in a statement. “This is the second time in six months that we have had to use the ‘high’ alert system and shows just why air pollution is a public health crisis,” he added.” [42] “President Donald Trump repeated his claim this week that Democratic leaders in California deserve blame for the fires, having failed to clear leaves and dead trees from forest floors. Wally Covington, professor of forestry at Northern Arizona University, agreed that forests have become overgrown and need to be thinned, but "not with a lawn rake."”[38]
Sustainable	A proactive approach to mitigate and adapt to climate change to build resilience [37]	

1. Solutions targeted

Any reference to where solutions are targeted.

**Table:** 

Solution target	Description	Example.
Government led	both local and national government action	Governments must prepare for heat waves, droughts, flooding and coastal storm surges “city has recently hired its first forestry officer, and announced a goal of planting 90,000 shade trees by 2021″ [10] “The tragedy at Kharghar, in which 14 people died from heat-related illness after attending an open air event, highlights the need for the local government to pay more attention to heat risk” [10]
Integrated solution	Public/community and institutions should act together	“Increasing public awareness is also critical, so individuals and communities get a better understanding of heat stress and modify their behaviour, said Pillai.For instance, said IIPH's Malvankar, communities could be more careful whether they should hold a wedding or religious gathering on hot summer days”[43] “Friends of the Earth said the government should go further still, to have a hope of halting the public health crisis from air pollution, which claims tens of thousands of lives a year and affects the quality of life of many more.”[43]
Specialized and external	medical practitioners, healthcare system, science and technology, and global cooperation	“Dr. Dominici added that this research could make clinicians and parents more aware of the range of disorders that affect children during hotter temperatures. ''If we know what types of diseases might be exacerbated on these days in kids, we can either prevent these diseases or when kids come into the E.D. clinicians are knowledgeable about what's happening.'” [44]

## CRediT authorship contribution statement

**Rabia Qusien:** Writing – review & editing, Writing – original draft, Visualization, Software, Project administration, Methodology, Investigation, Formal analysis, Data curation, Conceptualization.

## Declaration of competing interest

The authors declare that they have no known competing financial interests or personal relationships that could have appeared to influence the work reported in this paper.

## Footnotes

1 The researcher has trained a research assistant to code a small sample using the codebook to determine intercoder reliability.
